# Application of Texture Analysis in Diagnosis of Multiple Sclerosis by Magnetic Resonance Imaging

**DOI:** 10.5539/gjhs.v7n6p68

**Published:** 2015-03-30

**Authors:** Ali Abbasian Ardakani, Akbar Gharbali, Yalda Saniei, Arash Mosarrezaii, Surena Nazarbaghi

**Affiliations:** 1 MSc Student in Medical Physics, Medical Faculty, Urmia University of Medical Sciences, Urmia, Iran; 2 Medical Physics Department, Medical Faculty, Urmia University of Medical Sciences, Urmia, Iran; 3 Radiology Department, Medical Faculty, Imam Khomeini Hospital-Urmia University of Medical Sciences, Urmia, Iran; 4 Neurology Department, Medical Faculty, Urmia University of Medical Sciences, Urmia, Iran; 5 Neurology Department, Medical Faculty, Imam Khomeini Hospital-Urmia University of Medical Sciences, Urmia, Iran

**Keywords:** multiple sclerosis, magnetic resonance imaging, classification, diagnosis, computer-assisted, artificial intelligence

## Abstract

**Introduction:**

Visual inspection by magnetic resonance (MR) images cannot detect microscopic tissue changes occurring in MS in normal appearing white matter (NAWM) and may be perceived by the human eye as having the same texture as normal white matter (NWM). The aim of the study was to evaluate computer aided diagnosis (CAD) system using texture analysis (TA) in MR images to improve accuracy in identification of subtle differences in brain tissue structure.

**Material and Methods:**

The MR image database comprised 50 MS patients and 50 healthy subjects. Up to 270 statistical texture features extract as descriptors for each region of interest. The feature reduction methods used were the Fisher method, the lowest probability of classification error and average correlation coefficients (POE+ACC) method and the fusion Fisher plus the POE+ACC (FFPA) to select the best, most effective features to differentiate between MS lesions, NWM and NAWM. The features parameters were used for texture analysis with principle component analysis (PCA) and linear discriminant analysis (LDA). Then first nearest-neighbour (1-NN) classifier was used for features resulting from PCA and LDA. Receiver operating characteristic (ROC) curve analysis was used to examine the performance of TA methods.

**Results:**

The highest performance for discrimination between MS lesions, NAWM and NWM was recorded for FFPA feature parameters using LDA; this method showed 100% sensitivity, specificity and accuracy and an area of *A_z_* = 1 under the ROC curve.

**Conclusion:**

TA is a reliable method with the potential for effective use in MR imaging for the diagnosis and prediction of MS.

## 1. Introduction

Multiple sclerosis (MS) is a common chronic disorder of the central nervous system characterized by progressive degeneration and destruction of myelin (Compston & Coles, 2002). Diagnostic evaluation of MS can be difficult and must be performed by a specialized neurologist in treating MS. Magnetic resonance imaging (MRI) has been the most frequently-used technique since the 1980s for evaluating MS lesions within the brain and spinal cord and to monitor its progress (Thompson et al., 2000; Young et al., 1981). Conventional MRI is not adequate for detection of microscopic tissue changes in normal appearing white matter (NAWM) (Whiting et al., 2006). Recent MRI studies have shown that the measurement of the volume of brain and focal lesions, diffusion weighted imaging–derived parameters and magnetic transfer ratio techniques can provide more pathologically specific information when diagnosing MS (Bakshi et al., 2008; Rovira & León, 2008).

Structural abnormalities in medical imaging can be extracted by visual inspection, but complex patterns of pathology are difficult to interpret. Recent demand for a quantitative approach has rapidly increased. Since humans usually assess texture qualitatively, computerized texture analysis (TA) can increase the accuracy of assessment. Texture has different grey-level values, brightness, coarseness and colour across the image (Materka, 2004; Materka & Strzelecki, 1998). Tissues from normal brain and MS lesion have different texture features. At times, patterns within an image may be different, but are perceived by the human eye as having the same texture. Texture analysis detects pathological changes that cannot be perceived by the human eye using conventional MRI techniques. This mathematical technique increases quantification of and information about macroscopic MS lesions in the brain that are usually undetectable using conventional measurement of lesion volume and number (Fazekas et al., 1999). Recent studies have employed TA to assess MS lesion to differentiate between lesions, normal white matter (NWM) and NAWM and to monitor the progression of MS. The present study provides additional information to support this method.

Studies have attempted to classify MS lesions, NWM and NAWM. Zhang et al. (Zhang, Tong, Wang, & Li, 2008) used TA to classify MS lesions, NWM and NAWM. They showed that a combined set of texture features made it easier to distinguish MS lesions from NWM and NAWM. They also concluded that texture features were not successful for discriminating NWM from NAWM. Harrison et al. (Harrison et al., 2010) indicated that TA can be effective in classifying MS lesions from NWM and NAWM at 96%-100% accuracy. Tozer et al. (Tozer, Marongiu, Swanton, Thompson, & Miller, 2009) extracted texture features from magnetization transfer MRIs for normal controls and subjects with either clinically isolated syndrome (CIS) or MS. The texture features were compared between groups and it was found that there were no significant differences between the control and CIS subjects, but that parameters differed between MS subjects and the other groups. Mathias et al. (Mathias, Tofts, & Losseff, 1999) found significant differences in texture features between a normal control and a MS patient and a significant correlation between texture features and disability of the spinal cord. Multi-scale amplitude modulation–frequency modulation (AM–FM) texture analysis (Murray, Pattichis, Barriga, & Soliz, 2012) was used to evaluate the texture in multiple frequency scales. In this regard, Loizou et al. (Loizou et al., 2011) employed AM–FM features to differentiate between NWM, NAWM, and brain lesions at 0 and 6–12 months. Their findings indicated that there were significant differences in the AM–FM features between the groups.

The present study used TA to evaluate texture features extracted from MR images to differentiate between MS lesions, NWM and NAWM and to classify the different tissues. The most important texture features in TA are computed from statistical, model-base, structural and transform methods. texture features are analyzed coming from six main categories in the proposed computer-aided diagnosis (CAD) system: Histogram (statistical class), Absolute gradient (statistical class), Run-length matrix (statistical class), Co-occurrence matrix (statistical class), Auto-Regressive (AR) model (model class) and Wavelets (transform class) (Castellano, Bonilha, Li, & Cendes, 2004; Materka, 2004).

## 2. Materials and Methods

Fifty patients (22 male and 28 females), aged 34.7 ± 6.1 (mean age ± standard deviation) with a clinically definite MS and Fifty healthy subjects (24 males and 26 females) aged 37.5 ± 7.6 were recruited in the normal control group.

T2-weighted MR images of the patient and healthy subjects were acquired from a 1.5-T scanner (Philips Achieva; Philips Medical Systems, Best, The Netherlands) using a turbo spin echo sequence [TR=2000 ms, TE=100 ms, number of excitation (NEX)=3, matrix=512*512, field of view (FOV)=24cm, slice thickness=5 mm and inter slice gap=0.5 mm].

All MRI-detectable MS lesions were identified and placed with the help of an expert MS neurologist and confirmed by a radiologist. Five criteria were used to select the region of interest (ROI):

- One ROI was selected for each lesion/patient
- ROIs of NWMs, NAWMs and MS lesions were similar in shape and size
- ROIs of NWMs were selected from healthy subjects in the same location as those of MS lesions from MS patients
- NAWMs were selected that were adjacent to a MS lesion
- Only lesions size larger than of 100 pixels were used

One MR image/patient was input in MaZda software (version 4.6; The Technical University of Lodz, Institute of Electronics) for TA. More than 150 ROIs (50 MS lesions, 50 NWM, 50 NAWM) were selected for discrimination and classification. Up to 270 texture features extracted based on Histogram, Absolute gradient (spatial variation of grey-level values), Run-length matrix (counts of pixel runs with the specified gray-scale value and length in a given direction), Co-occurrence matrix (information about the distribution of pairs of pixels separated by given distance and direction), Auto-regressive model (description of correlation between neighbouring pixels) and Wavelets (decomposition image frequency at different scales) (Castellano et al., 2004; Materka, 2004).

Not all 270 texture features (parameters) were suitable or effective for use differentiating MS lesions, NAWMs and NWMs. Two reduction algorithms (Fisher and lowest probability of classification error and average correlation coefficients (POE+ACC)) were employed to reduce the parameters to the best 10 texture features showing the best discrimination between MS lesions, NAWMs and NWMs (Mucciardi & Gose, 1971).

Fisher algorithm selected up to ten features, with the highest being a ratio of between-class variance (D) to within-class variance (V). A POE+ACC algorithm produced set up ten features with minimization probability of classification error (POE) and average correlation coefficients (ACC) between features. In brief, the POE+ACC algorithm introduces ten features with high discriminatory potential and a least correlation with features that are already selected.

Each feature reduction method was applied equally the MS lesions, NAWMs and NWMs to find the best 10 texture features. The fused Fisher and POE+ACC (FFPA) texture features were compared to the separate Fisher and POE+ACC features to evaluate which method provides better accuracy for classification. Before analysis the features were standardized as follows”


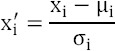


where 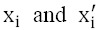 are feature values before and after standardization, respectively, μ_i_ is the mean and σ_i_ is the standard deviation of the i^th^ feature. These features were analysed using both standard and nonstandard states.

**Figure 1:**
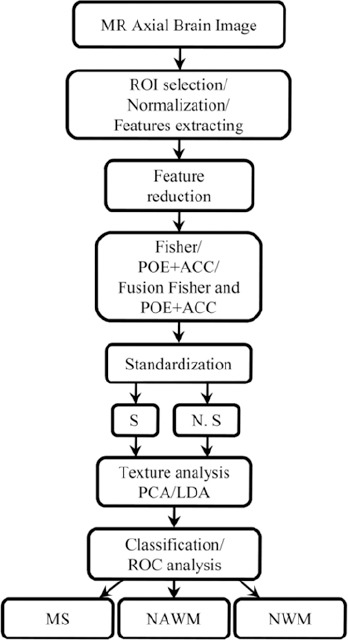
Overview of general texture analysis process in the MR brain image

Principle component analysis (PCA) and linear discriminant analysis (LDA) were used to investigate the features and transform the data to lower-dimensional spaces (Fukunaga, 1990; Webb, 2003). The K-NN (K=1) classifier was used for features resulting from PCA and LDA (Anderson & Rosenfeld, 1993). Classification was carried for MS lesions versus NWM, MS lesions versus NAWM, and NWM versus NAWM. Receiver operating characteristic (ROC) curve analysis was employed to compare the discrimination performance of the TA methods using the area under the ROC curve (*A_z_*) (Van Erkel & Pattynama, 1998). In addition, six objectives that indices sensitivity (SEN), specificity (SPC), overall accuracy (ACC), positive predictive value (PPV) and negative predictive value (NPV)are also applied to assess the performance of the proposed methods. ROC analysis was performed with the SPSS software (SPSS Inc., Chicago, USA). Figure 1 shows the steps of CAD processing.

## 3. Results

Twelve options for texture analysis were utilized: three feature reduction methods (Fisher, POE+ACC and FFPA), two TA methods (PCA and LDA) and two standardization states (standardization and non-standardization). A total of 100 cases (50 patients with MS and 50 healthy subjects) were selected to evaluate the classification accuracy of the proposed method.

**Figure 2:**
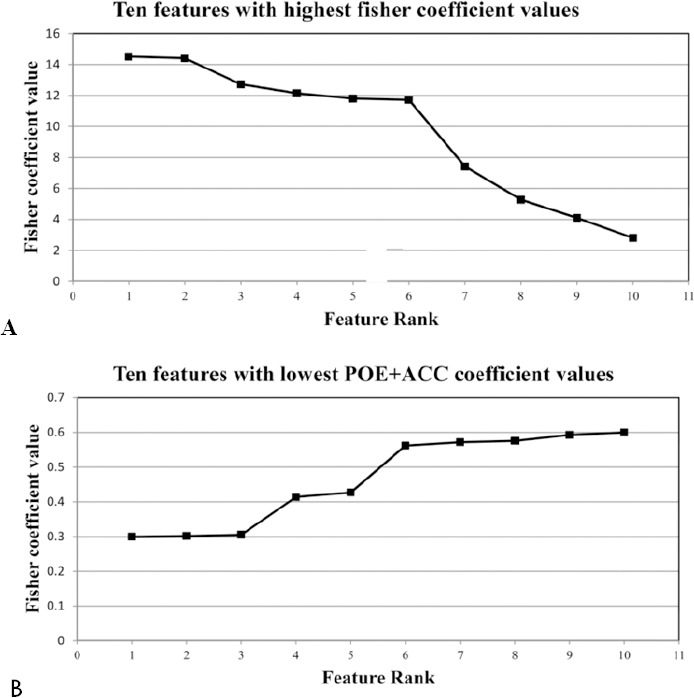
Evolution of two reduction methods for texture analysis. A) Fisher coefficient with 10 highest values, B) POE+ACC coefficient with 10 lowest values

Figure 2(a) and Table 1 show the best 10 features with the highest Fisher coefficient values. Grey level non-uniformity in the 45 degree (45dgr_GLevNonU) and 135degree (135dr_GLevNonU) directions had the highest Fisher coefficients. The other Fisher features were mostly from: percentile 90% (Perc.90%), percentile 99% from histogram; S(2, -2) Sum Average (S(2,-2)SumAverg) and S(2, 0) Sum Average (S(2, 0)SumAverg) from Co-occurrence matrix where S(i, j) shows the direction of matrix construction and inter pixel distance i along the rows and j along the columns of matrix; Energy of wavelet coefficient in “low-low” energy components in first levels wavelet decomposition (WavEnLL_s-1), “low-low” energy components in second levels wavelet decomposition (WavEnLL_s-2), “high-low” energy components in first levels wavelet decomposition (WavEnHL_s-1) and “low-high” energy components in first levels wavelet decomposition (WavEnLH_s-1).

**Table 1:** Summary of best ten Fisher features with highest values

Feature rank	Feature	Feature Group	Fisher coefficient value
**1**	45dgr_GLevNonU	Run-length matrix	14.5109
**2**	135dr_GLevNonU	Run-length matrix	14.4105
**3**	Perc.90%	Histogram	12.7285
**4**	Perc.99%	Histogram	12.1486
**5**	S(2,-2)SumAverg	Co-occurrence matrix	11.8077
**6**	S(2,0)SumAverg	Co-occurrence matrix	11.7172
**7**	WavEnLL_s-1	Wavelet	7.4151
**8**	WavEnLL_s-2	Wavelet	5.2741
**9**	WavEnHL_s-1	Wavelet	4.0968
**10**	WavEnLH_s-1	Wavelet	2.8063

Figure 2(b) and Table 2 show the best 10 features with the lowest POE+ACC values. There were six parameters common to Fisher and POE+ACC reduction methods. The other POE+ACC features were mostly from: three vectors of Autoregressive model parameters (Teta 1, Teta 3 and Teta 4) and Run Length Non-uniformity in 135 degree direction (135dr_RLNonUni). Desirable texture features are those with the highest Fisher and/or lowest POE+ACC coefficients show the best discrimination between MS lesions, NWMs and NAWMs.

**Table 2:** Summary of best ten POE+ACC features with lowest values

Feature rank	Feature	Feature Group	POE+ACC coefficient value
**1**	WavEnHL_s-1	Wavelet	0.2998
**2**	Perc.90%	Histogram	0.3017
**3**	Teta1	Autoregressive model	0.3053
**4**	WavEnLH_s-1	Wavelet	0.414
**5**	Teta4	Autoregressive model	0.4269
**6**	S(2,-2)SumAverg	Co-occurrence matrix	0.5622
**7**	135dr_RLNonUni	Run-length matrix	0.5719
**8**	S(2,0)SumAverg	Co-occurrence matrix	0.5757
**9**	Teta3	Autoregressive model	0.5928
**10**	Perc.99%	Histogram	0.6

The diagnostic performance of the texture analysis methods are shown in Tables 3, 4 and 5. The features extracted by the Fisher algorithm are shown in Table 3. The features analysed using LDA were found to have higher discriminative power than PCA for all three groups. They had 100% sensitivity, specificity, accuracy, PPV and NPV for MS lesions versus NWM (or NAWM) and 98% sensitivity, 100% specificity, 99% accuracy, 100% PPV and 98.04% NPV for NAWM versus NWM.

**Table 3:** Summary of performance for different groups and Fisher feature reduction method

Group	Method of feature analysis	SEN(%)	SPC(%)	ACC(%)	PPV(%)	NPV(%)	*A_z_* value	Correct classification
MS vs. NWM	NS.PCA	98	98	98	98	98	0.989	**98% (98/100)**
	S.PCA	100	100	100	100	100	1	**100% (100/100)**
	NS.LDA	100	100	100	100	100	1	**100% (100/100)**
	S.LDA	100	100	100	100	100	1	**100% (100/100)**
MS vs. NAWM	NS.PCA	88	90	89	88.23	89.9	0.891	**81% (81/100)**
	S.PCA	96	96	96	96	96	0.962	**96% (96/100)**
	NS. LDA	100	100	100	100	100	1	**100% (100/100)**
	S.LDA	100	100	100	100	100	1	**100% (100/100)**
NWM vs. NAWM	NS.PCA	58	60	59	59.18	58.82	0.587	**59% (59/100)**
	S.PCA	96	100	98	100	96.15	0.976	**98% (98/100)**
	NS.LDA	98	100	99	100	98.04	0.994	**99% (99/100)**
	S.LDA	98	100	99	100	98.04	0.994	**99% (99/100)**

SEN = sensitivity; SPC = specificity; ACC = accuracy; PPV = positive predictive value; NPV = negative predictive value; *A_Z_*= area under ROC curve.

**Table 4:** Summary of performance for different groups and POE+ACC feature reduction method

Group	Method of feature analysis	SEN(%)	SPC(%)	ACC(%)	PPV(%)	NPV(%)	*A_z_* value	Correct classification
MS vs. NWM	NS.PCA	100	100	100	100	100	1	**100% (100/100)**
	S.PCA	100	100	100	100	100	1	**100% (100/100)**
	NS.LDA	100	100	100	100	100	1	**100% (100/100)**
	S.LDA	100	100	100	100	100	1	**100% (100/100)**
MS vs. NAWM	NS.PCA	96	98	97	97.96	96.08	0.978	**97% (97/100)**
	S.PCA	100	94	97	94.33	100	.982	**97% (97/100)**
	NS.LDA	96	96	96	96	96	0.967	**96% (96/100)**
	S.LDA	96	96	96	96	96	0.967	**96% (96/100)**
NWM vs. NAWM	NS.PCA	92	98	95	97.87	92.45	0.951	**95% (95/100)**
	S.PCA	100	98	99	98.04	100	.995	**99% (99/100)**
	NS.LDA	98	98	98	98	98	0.99	**98% (98/100)**
	S.LDA	98	98	98	98	98	0.99	**98% (98/100)**

SEN = sensitivity; SPC = specificity; ACC = accuracy; PPV = positive predictive value; NPV = negative predictive value; *A_z_*= area under ROC curve.

**Table 5:** Summary of performance for different groups and fusion Fisher and POE+ACC (FFPA) feature reduction method

Group	Method of feature analysis	SEN(%)	SPC(%)	ACC(%)	PPV(%)	NPV(%)	*A_z_* value	Correct classification
MS vs. NWM	NS.PCA	98	98	98	98	98	0.989	**98% (98/100)**
	S.PCA	100	100	100	100	100	1	**100% (100/100)**
	NS.LDA	100	100	100	100	100	1	**100% (100/100)**
	S.LDA	100	100	100	100	100	1	**100% (100/100)**
MS vs. NAWM	NS.PCA	88	90	89	89.8	88.23	0.891	**89% (89/100)**
	S.PCA	96	94	95	94.12	95.92	0.971	**95% (95/100)**
	NS.LDA	100	100	100	100	100	1	**100% (100/100)**
	S.LDA	100	100	100	100	100	1	**100% (100/100)**
NWM vs. NAWM	NS.PCA	58	60	59	49.15	50.85	0.587	**59% (59/100)**
	S.PCA	98	100	99	100	98.04	0.994	**99% (99/100)**
	NS.LDA	100	100	100	100	100	1	**100% (100/100)**
	S.LDA	100	100	100	100	100	1	**100% (100/100)**

SEN = sensitivity; SPC = specificity; ACC = accuracy; PPV = positive predictive value; NPV = negative predictive value; *A_z_*= area under ROC curve.

Table 4 shows that POE+ACC features performed similarly in PCA and LDA for MS lesions versus NWM with 100% sensitivity, specificity, accuracy, PPV, and NPV. The standardization PCA (S.PCA) outcomes for LDA for MS versus NAWM had 100% sensitivity, 94% specificity, 97% accuracy, 94.33% PPV and 100% NPV. S.PCA better classified NAWM and NWM and recorded 100% sensitivity, 98% specificity, 99% accuracy, 98.04% PPV and 100% NPV.

For the FFPA features set, texture classification with LDA was 100% between all 3 pair groups for sensitivity, specificity, accuracy, PPV and NPV. PCA showed large fluctuation in classification (Table 5).

**Figure 3:**
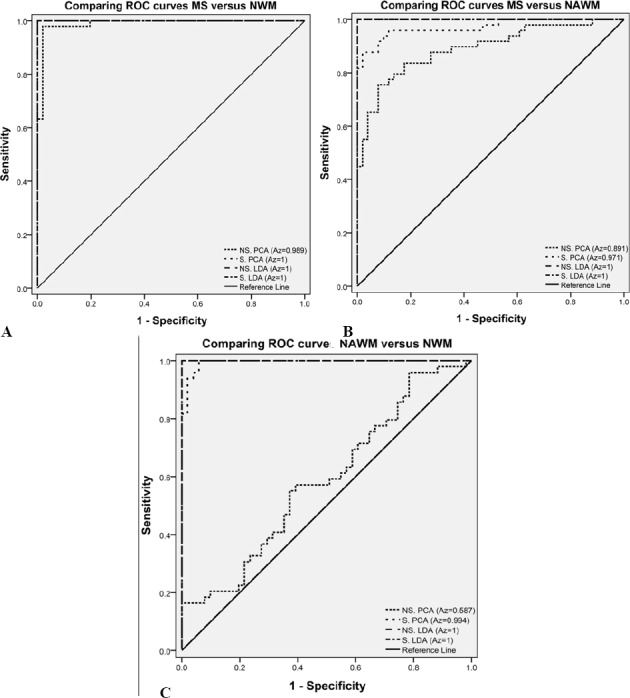
The diagram of the ROC curve for texture analysis in FFPA feature reduction method. A) MS vs. NWM, B) MS vs. NAWM, C) NAWM vs. NWM

The ROC curves for LDA and PCA in FFPA features for the proposed CAD system are plotted in Figures 3(a) to 3(c). As shown, LDA showed the best performance for classification of MS lesions versus NWM (or NAWM) and NWM versus NAWM at *A_z_* = 1.

## 4. Discussion

The primary objective of this study was to differentiate between NAWM and NWM using MR imaging to develop a more effective method of predicting MS. The three feature reduction elimination methods, two standardization state and two texture data analysis methods provided 12 states per ROI case study.

**Figure 4:**
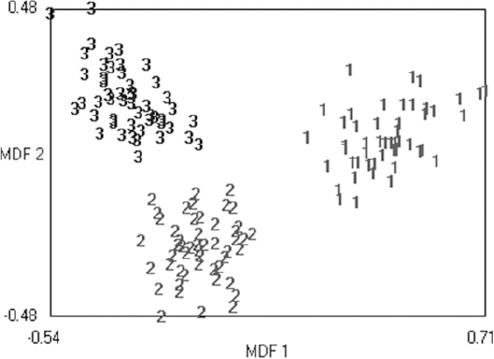
Sample distributions after LDA texture analysis methods. MDF: Most discriminating features; 1: MS lesion, 2: NAWM, 3: NWM

The results show that TA differentiated MS lesions from NWM (or NAWM) and NAWM from NWM with high accuracy. The best results were derived using FFPA features with LDA and showed 100% sensitivity, specificity, accuracy, PPV and NPV for MS lesions versus NWM and NAWM (Table 5). Figure 3 shows that LDA had the best performance in all 3 states at *A_z_* = 1. Figure 4 shows the discrimination distributions for the best results using LDA. As seen, LDA had the greatest power of discrimination between the MS lesion, NAWM and NWM tissue types.

The *A_z_* value for PCA indicates the POE+ACC features had the best results. In LDA, FFPA features showed excellent accuracy for all three tissue pairs. The Fisher method selected 10 features based on the difference between textures and POE+ACC minimized the number of errors in sample classification. Since the criteria for texture selection by Fisher and POE+ACC are different, it can be said that these two methods are complementary. The feature reduction methods were also fused and showed improved performance for the proposed CAD system.

Under all conditions, feature standardization only affected PCA and improved performance. The largest effect was for FFPA features for classification of NAWM and NWM. The *A_z_* values for NS.PCA versus S.PCA were 0.587 vs. 0.994, respectively. It had no impact on LDA.

Several studies have evaluated texture features for differentiation of a control from MS subjects. Harrison et al. (Harrison et al., 2010), used combination features based on co-occurrence matrix, wavelet, gradient, autoregressive models and histograms to correctly classify NWM versus NAWM (85%) and MS lesions versus NWM (or NAWM) (100%). Zhang et al. (Zhang et al., 2008) compared the grey-level co-occurrence matrix (GLCM) and derived combinations of 24 texture features derived from GLCM, run-length matrix, gradient, autoregressive model and wavelet. They found that the classification accuracy of combined sets of texture features was better than GLCM when discriminating MS lesions and NWM (combined versus GLCM: 100% versus 92.67%), equally discriminated MS lesions versus NAWM (100% vs. 100%) and between all three groups (88.89% vs. 88.89%), but was less effective at discriminating NWM and NAWM (58.33% vs. 66.67%).

Magnetization transfer imaging can quantify and decrease demyelination in white matter (Dousset et al., 1992; Schmierer, Scaravilli, Altmann, Barker, & Miller, 2004). Tozer et al. (2009) showed that the magnetization transfer ratio (MTR) parameters were effective for MS. They extracted texture features based on GLCM from MTR scans from 23 healthy controls, 32 MS patients and 38 CIS patients. They failed to differentiation between healthy and CIS groups but texture features could differ between MS and the other groups.

The present study on TA for MS indicates that the proposed method was useful for differentiation between MS lesions, NWM and NAWM. Since TA can detect non-visible microstructural changes in tissue, it may be effective for early diagnosis and treatment of MS.

The study had some limitations. First, the data group was small; further investigation with a larger data set is needed. Second, feature combination tools were not available for MaZda. For example, averaging Run-length matrix features for four orientations was hard to perform with MaZda. Third, the position of the subjects during the image acquisition was different. Since the magnetic field is non-uniform along the MRI system magnet, textural features of the tissue may show differences.

## 5. Conclusion

The main advantage of this method is that it can be used as an auxiliary tool to improve accuracy of diagnosis of MS and it requires no additional time or cost. These comparative results showed that the proposed CAD system has the potential to characterize and classify MS and can be effective for prediction of MS lesions and to identify appropriate therapies.
